# Long-Term Parental Unemployment in Childhood and Subsequent Chronic Fatigue Syndrome

**DOI:** 10.5402/2013/978250

**Published:** 2013-01-27

**Authors:** Esme Fuller-Thomson, Rukshan Mehta, Joanne Sulman

**Affiliations:** ^1^Factor-Inwentash Faculty of Social Work, University of Toronto, 246 Bloor Street West, Toronto, ON, Canada M5S 1A1; ^2^Department of Family and Community Medicine, University of Toronto, Toronto, ON, Canada M5G 1V7; ^3^Theodore J. Freedman Department of Social Work, Mount Sinai Hospital, Toronto, ON, Canada M5G 1X5

## Abstract

*Objective*. The association between long-term parental unemployment in childhood and chronic fatigue syndrome was examined in a population-based sample of women. *Methods*. A secondary analysis of data from a regionally representative sample of women (*n* = 7, 288) from the Canadian Community Health Survey (2005) was conducted using logistic regression. Age and race as well as the following clusters of factors were controlled for: (1) other childhood adversities, (2) adult health behaviors and hypertension, (3) adult stressors and socioeconomic status, and (4) adult mental health. *Results*. When adjusting for age and race only, the odds ratio of chronic fatigue syndrome among those reporting parental unemployment was 4.12 (95% CI: 2.60, 6.52) compared to those not reporting parental unemployment. When controlling for age and race plus all four clusters of factors the odds ratio for chronic fatigue syndrome dropped slightly to 3.05 (95% CI: 1.81, 5.14), but remained statistically significant. *Conclusions*. This study provides evidence for a significant association between long-term parental unemployment in childhood and chronic fatigue syndrome even after controlling for a wide range of potential risk factors.

## 1. Introduction

Chronic fatigue syndrome (CFS) is characterized by severe and disabling fatigue in addition to other disturbances in concentration, short-term memory, sleep disruption, and musculoskeletal pain [1]. Community prevalence rates of CFS range between 0.2% and 2.6% [2]. In the United States, CFS affects between 400,000 and 800,000 people. Women are between 1.3 and 1.7 times more likely to be diagnosed with the syndrome than men [3]. CFS patients are heavy users of health services and almost every family doctor has patients with CFS. In the United States, CFS results in an annual cost of $9.1 billion due to medical expenses and lost productivity [2].

Little is known about the etiology of CFS [4]. Several studies suggest that serious childhood stressors such as physical abuse, parental addictions and lower socioeconomic status increase an individual's vulnerability to developing CFS. One factor that, to our knowledge, has not been examined as a risk factor for CFS is parental unemployment.

There is considerable evidence to suggest that parental unemployment is associated with a wide range of negative educational, social, health behaviors, and mental health outcomes for children, adolescents, and adults [5, 6]. Parental unemployment is associated with poorer school performance and negative health outcomes among children and adolescents. Children of unemployed parents are more likely to report being bullied and socially isolated at school, and to experience multiple changes of schools [5]. This may significantly impact learning, as children of unemployed parents have poorer grades and drop out of school earlier than their peers with employed parents [5]. In cases where unemployment leads to extreme poverty, children are vulnerable to food insecurity and nutritional problems which may also influence school performance [7]. Young children with unemployed parents appear to be more susceptible to psychosomatic symptoms such as headaches, stomach-aches, and problems with eating and sleeping [8]. In comparison to children of employed parents, those with parents who are unemployed are more likely to have accidents and to be hospitalized [8]. In addition to unintentional injuries, unemployed fathers are three times more likely to abuse their children than employed fathers [8].

Parental unemployment is also associated with lower levels of self-esteem and higher levels of anxiety and mental illness in children and adolescents [9]. The strong association between parental unemployment and child and adolescent depression, suicidal ideation, self-harming behaviors, and suicide has been well established [10]. Christoffersen suggests that the increased risk of suicide is due to the loss of family support caused by the upheaval of unemployment [9]. Other factors such as depression, anxiety and substance abuse and the physical and sexual abuse that sometimes accompanies parental unemployment may also influence children's suicidal behaviour [9]. The outcomes of parental unemployment also depend on the salience of unemployment for parents. Many unemployed parents experience considerable financial strain, anxiety, depression, and addictions [11]. These factors in turn may result in increased parental conflict, higher rates of divorce, and poorer parenting behaviors exacerbating mental health concerns in children [5].

A few panel studies have followed the children of unemployed and employed parents into later adolescence and adulthood to document the long-term social and educational outcomes of parental unemployment [5, 9]. These studies indicate that the children of unemployed parents are more likely to leave high school before graduation, to experience higher rates of unemployment themselves (particularly true of men), and to have lower incomes than their peers who did not experience parental unemployment [5, 9]. According to Flouri and Buchanan, these children are not exposed to “working role models” in the home and may not develop the necessary behavioral correlates of work [12]. Parental unemployment has been demonstrated to reduce social networks for youth and adults thereby decreasing social capital [13].

The literature examining the long-term outcomes of childhood parental unemployment on mental health and health is limited. Kaltiala-Heino and colleagues report that children who have experienced parental unemployment are more likely to experience depression in adulthood [14]. Adult onset anxiety disorders may also be associated with parental unemployment early in life [15].

Although negative health behaviors such as smoking are associated with parental unemployment in childhood [16], surprisingly little is known about links between parental unemployment and physical health. This gap in the literature extends to the relationship between parental unemployment and subsequent chronic fatigue syndrome. Other adverse childhood experiences, such as physical abuse and parental addictions, have been identified as important risk factors for chronic fatigue syndrome [17].

In this study we examine the association between parental unemployment and subsequent chronic fatigue syndrome, while controlling for a wide range of possible mediating factors including demographics, other childhood adversities, adult stressors and socioeconomic status, adult health behaviors and hypertension, and mental health.

### 1.1. Other Childhood Adversities

Childhood parental unemployment often occurs with or exacerbates the presence of poverty, physical abuse, sexual abuse, parental mental illness, parental addictions, parental conflict, divorce, step-family situations, and single parent families. A number of adverse childhood experiences have been implicated in the development of chronic fatigue syndrome in adulthood [17]. Known risk factors for CFS include poverty, childhood physical and sexual abuse, and low socioeconomic status.

### 1.2. Adult Health Behaviors and Hypertension

Parental unemployment has been associated with adult health behaviors such as smoking [18]. In women, childhood socioeconomic status plays influences smoking behaviors in adulthood [19]. Chronic fatigue syndrome has also been associated with adverse health behaviors such as tobacco use [20]. Kennedy and colleagues report some emerging studies suggesting that obesity and hypertension may be additional factors that impact the development of CFS [21].

### 1.3. Adult Stressors and Socioeconomic Status

As documented earlier, childhood experiences of parental unemployment influence adult education, lower household income, job status, daily stress, and divorce [5, 18]. Marital status is used as a proxy for social support and has also been identified as an important factor in determining severity and functional impairment in CFS patients [22]. Clinical studies indicate that individuals suffering from CFS disproportionately have lower socioeconomic status. Jason and colleagues in a community-based study of CFS found those with lower levels of education and occupational status had the highest prevalence of CFS [23].

### 1.4. Mental Health

The long-term impacts of parental unemployment on mental health include but are not limited to depression, anxiety, and lower self-esteem [14–16]. In turn, the prevalence of chronic fatigue syndrome is markedly higher among those with anxiety disorders and depression [16]. Reports suggest that individuals who suffer from CFS also have a high prevalence of trauma and stressful life events [16].

It is important to understand the relationship between parental unemployment and CFS in the light of the present economic climate. There are few studies that have examined the long-term health outcomes of childhood parental unemployment. To our knowledge, no studies have examined the link between childhood parental unemployment and CFS. Using a large regionally representative community sample, this study seeks to examine the association between parental unemployment and CFS in adult women, while controlling for risk factors including demographics, other childhood adversities, adult health behaviors and hypertension, adult stressors and SES and mental health.

## 2. Materials and Methods

### 2.1. Data Source and Sample

We conducted a secondary data analysis of female respondents from the Manitoba and Saskatchewan sample of the 2005 Canadian Community Health Survey (*n* = 7, 244). The response rate was 84%. Respondents were asked whether, as a “child or a teenager,” their “father or mother (did) not have a job for a long time when they wanted to be working?” (666 reported yes).

Individuals were also asked to indicate whether they had “long-term conditions lasting six months or more” “that were diagnosed by a health professional.” Specifically they were asked “Do you have chronic fatigue syndrome?” (*n* = 99 reported yes).

### 2.2. Statistical Analysis

We conducted six logistical regression analyses using chronic fatigue syndrome as the dependent variable. The decision about which variables to include was hypothesis driven, based on our review of the literature. In our first and all subsequent models, we controlled for age and race. In the second model we controlled for the number of other childhood adversities including childhood physical abuse, parental divorce, and parental addictions. The third model included adult health behaviours such as obesity, smoking, and alcohol use. In the fourth model we controlled for adult stress and socioeconomic status, which included factors such as self-reported stress, education, and income and marital status (a proxy for social support). The fifth model controlled for mental health conditions that were indicated through self-report of a medical diagnosis of (1) mood disorders and (2) anxiety disorders. Finally we adjusted for all of the above-mentioned risk factors in our last model.

**Figure 1:**
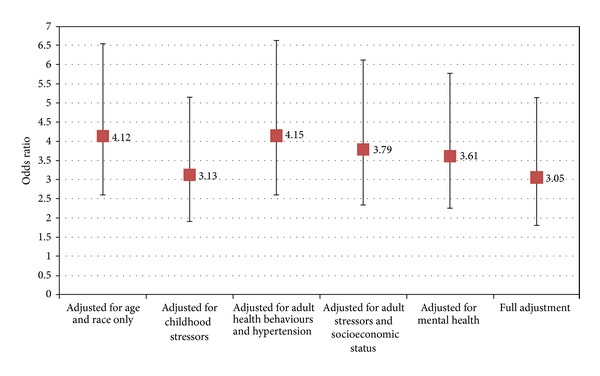
Odds ratio and 95% confidence interval of chronic fatigue syndrome for women reporting parental unemployment during respondent's childhood versus individuals reporting parents not unemployed. All data are adjusted for age and race. Sample sizes vary from *n* = 7,193 in the first model to *n* = 7,088 in the fully adjusted model. Source: Canadian Community Health Survey, 2005.

Figure 1 illustrates the odds ratios associated with our key variable of interest: parental unemployment. This figure presents the association between parental unemployment and CFS varied when we controlled for each cluster of risk factors. Due to missing data for the independent variables, the sample sizes varied from *n* = 7, 193 in the age and race adjusted model to *n* = 7, 088 in the fully adjusted model. We used a weighting variable created by Statistics Canada to adjust for the probability of selection and nonresponse in all our models.

### 2.3. Measures

#### 2.3.1. Other Childhood Adversities

Respondents were asked if they had experienced the following stressors during childhood and adolescence: (1) “Were you ever physically abused by someone close to you?” (2) “Did your parents get a divorce?” (3) “Did either of your parents drink or use drugs so often that it caused problems for the family?” Respondents answers were summed to get the number of other childhood adversities. Response could range from 0 other adversities to 3 other adversities.

#### 2.3.2. Adult Health Behaviors and Hypertension

Body mass index (BMI) was divided into four categories (BMI < 25, overweight, obese, and missing data category). Smoking status was dichotomized as current or former smoker versus never smoked. Hypertension was based on the respondent's self-report that they had been diagnosed with high blood pressure by a health professional.

#### 2.3.3. Adult Stressors

Educational attainment, self-reported stress, marital status, and household income were examined under adult stressors. These stressors were categorized as following: educational attainment (<high school graduation, high school graduation, postsecondary graduation); daily stress (no stress to a bit of stress, quite a bit to extreme stress); marital status (not married, married); income (<$15,000, $15,000–$29,999, $30,000–$49,999; $50,000 or more, missing data category).

#### 2.3.4. Mental Health

The two mental health conditions of interest in our study were depression and anxiety disorders. Depression was assessed by self-report of mood disorders to questions. Women were asked (1) “Do you have a mood disorder such as depression, bipolar disorder, mania or dysthymia?” (2) “Do you have an anxiety disorder such as a phobia, obsessive compulsive disorder, or a panic disorder?”.

## 3. Results

We found a strong and significant association between long-term parental unemployment in childhood and chronic fatigue syndrome in women (see Figure 1). When we adjusted for age and race, we found the odds ratio for CFS among those women who reported parental unemployment to be 4.12 (95% CI: 2.60, 6.52) when compared to those who did not report parental unemployment. When we adjusted for number of other childhood adversities, the odds ratio for CFS among those reporting parental unemployment dropped substantially to 3.13 (95% CI: 1.91, 5.13). Adjustments for adult health behaviors and hypertension did not change the odds ratio when compared to the age and race adjusted model (OR = 4.15, 95% CI: 2.60, 6.62). The odds ratios for CFS among women who had experienced parental unemployment in the models controlling for adult stressors and socioeconomic status and adult mental health were relatively similar (OR = 3.79, 95% CI: 2.35, 6.1 and OR = 3.61, 95% CI: 2.26, 5.77). Finally when controlling for all the above risk factors, the odds ratio for CFS among women reporting parental unemployment dropped to 3.05 (95% CI: 1.81, 5.14), but remained statistically significant.

Table 1 shows the age-race adjusted model and the fully adjusted logistic analysis of chronic fatigue syndrome. In the fully adjusted model, in addition to parental unemployment five other characteristics were associated with CFS: White women had five times higher odds of CFS than did non-Whites. In comparison to those who had not finished high school, those who had finished high school had twice the odds of CFS. In comparison to respondents who had a household income of $50,000 or more, women who had an income of less than $15,000 had almost four times the odds of CFS and women with an income of $30,000–$49,999 had twice the odds. Those reporting more stress had three times the odds of CFS compared to women with lower levels of stress. Women with anxiety disorders had three times the odds of CFS in comparison to those who had not been diagnosed with anxiety disorders.

## 4. Discussion

This study examined the association between long-term parental unemployment and chronic fatigue syndrome when controlling for other childhood adversities, adult health behaviors, adult stressors, adult socioeconomic status, and adult mental health. We found a strong and significant association between parental unemployment and CFS after adjusting for these factors. Our analysis suggests that adjusting for number of other childhood adversities substantially reduced the parental unemployment-CFS relationship although parental unemployment was still significantly associated with CFS. In the literature, one childhood stressor in particular, physical abuse, has been implicated as an important risk factor for CFS [16, 24]. Albeit substantially reduced, the relationship between parental unemployment and CFS remains strong and significant after controlling for the number of other childhood adversities including physical abuse, parental divorce, and parental addictions.

Adult health behaviors and hypertension do not appear to affect the relationship between parental unemployment and CFS. Felitti and colleagues suggested that health behaviors were the major pathways that mediated the relationship between adverse childhood experiences such as parental unemployment and adult health conditions [25]. Adult health behaviors such as smoking, alcohol use, and drug abuse may be used as coping mechanisms in adulthood [25]. Although adult health behaviors have been associated with both CFS and parental unemployment, it appears that these variables do not influence the relationship directly.

Including adult stressors resulted in a slight decrease in the relationship between parental unemployment and chronic fatigue syndrome. We anticipated that parental unemployment would lead to lower socioeconomic status and greater distress in adulthood, which in turn would influence CFS. Christoffersen hypothesized that the relationship between parental unemployment and a child's eventual career trajectory may be mediated through self-esteem and self-image, thus impacting social and economic well-being in adulthood [5].

Mental health conditions such as mood and anxiety disorders have been strongly linked to both CFS and parental unemployment [9, 14, 26, 27]. These conditions seem to impact the relationship between parental unemployment and CFS. However the association between parental unemployment and CFS remained robust after controlling for both these mental health conditions.

To our knowledge this was the first study to examine the association between parental unemployment and CFS in a large population-based study of women. Even after controlling for the number of other childhood adversities, adult health behaviors and hypertension, adult stressors, socioeconomic status, and adult mental health, there remains a strong relationship between parental unemployment and CFS.

Future research would benefit from examination of the potential role that the hypothalamic-pituitary-adrenal (HPA) axis plays in the relationship between parental unemployment and CFS [28]. Although our research could not explore this issue, HPA axis dysfunction has been previously implicated in functional somatic syndromes such as CFS. There appears to be a strong and significant correlation between childhood adversity and the development of CFS [28]. Heim and her colleagues have examined the role of the HPA axis in influencing the relationship between another childhood stressor (i.e., abuse) and CFS [28]. They found early childhood adversity and, in particular, abuse early in life to be associated with CFS. They hypothesized that the relationship may be mediated through physiological pathways that cause hypocortisolism, which, in turn, may cause fatigue. Accordingly, they found that individuals who were abused in childhood had between 3 and 8 times the risk of developing CFS in comparison to those who were not abused. The degree of childhood trauma also increased the severity of CFS symptoms.

There are a number of limitations to this study. The CCHS survey is a cross-sectional study and therefore does not permit causal analysis of the data. Retrospective data was used to assess childhood parental unemployment; prospective studies would minimize the likelihood of differential recall among participants. Chronic fatigue syndrome status was based on the respondents' report of a diagnosis by a health professional. The chances of underreporting are high as research suggests that fewer than 20% of individuals with CFS have been diagnosed.

Childhood parental unemployment remained an independent risk factor for chronic fatigue syndrome even after adjustment for most of the known risk factors for CFS. It may therefore be useful for clinicians and researchers to assess patients exhibiting symptoms of CFS for childhood experiences of parental unemployment and other adverse childhood experiences. Future research should also examine the role of other potential risk factors in mediating the relationship between parental unemployment and CFS. The risk factors we examined in this study vary in their importance; however, childhood physical abuse appears to play an important role in development of CFS and should be examined further.

This study provides additional support to the emerging literature on the long-term mental and physical health consequences of parental unemployment. In conjunction with the substantial literature suggesting short-term negative outcomes for the well-being and mental and physical health of the children and adolescents of unemployed parents, the study underlines the urgency of adopting policies and programs to decrease unemployment levels and to provide an expanded social safety net for those who are out of work. The intergenerational transfer of the negative consequences of unemployment makes it imperative that as a nation we move quickly to improve employment opportunities.

## Figures and Tables

**Table 1:** Age-race adjusted logistic regression of chronic fatigue syndrome (*n* = 7,193) and fully adjusted odds ratio of chronic fatigue syndrome (*n* = 7,088) in a representative community sample. Source: Canadian Community Health Survey, 2005.

	Age-race adjusted odds ratios	Lower 95% confidence interval	Upper 95% confidence interval	Fully adjusted odds ratios	Lower 95% confidence interval	Upper 95% confidence interval
Parental unemployment						
Yes unemployment	**4.115**	2.598	6.518	**3.047**	1.807	5.139
No unemployment	1.00	Reference	Reference	1.00	Reference	Reference

Age by decade						
12–19	1.00	Reference	Reference	1.00	Reference	Reference
20–29	8.436	0.584	121.778	9.206	0.625	135.687
30–39	7.401	0.507	108.045	10.261	0.679	154.970
40–49	8.092	0.561	116.664	11.934	0.795	179.210
50–59	7.992	0.551	115.954	10.154	0.667	154.491
60–69	9.608	0.651	141.844	12.884	0.825	201.224
70–79	8.109	0.538	122.152	8.891	0.594	142.607
80+ years	5.614	0.342	92.087	6.987	0.407	120.004
Race (self-report)						
White	**4.895**	1.695	14.136	**5.419**	1.820	16.140
Non-White	1.00	Reference	Reference	1.00	Reference	Reference

Number of other childhood adversities				1.289	0.990	1.677

Body mass index						
Underweight or normal (bmi < 25)				1.00	Reference	Reference
Overweight				0.724	0.420	1.247
Obese				1.298	0.760	2.216
Missing				0.312	0.069	1.408
High blood pressure						
Yes				1.422	0.843	2.398
No				1.00	Reference	Reference
Smoking status						
Current or former				1.035	0.648	1.655
Never smoked				1.00	Reference	Reference

Education						
<High school graduate				1.00	Reference	Reference
High school grad or some postsecondary education				**2.155**	1.119	4.149
Postsecondary graduation				1.184	0.608	2.309
Household income						
No or <$15,000				**3.829**	1.810	8.102
$15,000–$29,999				1.378	0.634	2.992
$30,000–$49,999				**2.027**	1.095	3.750
$50,000 or more				1.00	Reference	Reference
Missing data				**3.017**	1.591	5.722
Stress level (self-report)						
Quite a bit to extremely stressed				**3.057**	1.965	4.754
No stress to a bit of stress				1.00	Reference	Reference
Marital status				0.744	0.456	1.217
Married						
Not married				1.00	Reference	Reference

Mood disorder						
Yes				1.298	0.702	2.400
No				1.00	Reference	Reference
Anxiety disorder						
Yes				**3.042**	1.685	5.491
No				1.00	Reference	Reference
